# Cec4-Derived Peptide Inhibits Planktonic and Biofilm-Associated Methicillin Resistant Staphylococcus epidermidis

**DOI:** 10.1128/spectrum.02409-22

**Published:** 2022-12-01

**Authors:** Chengju Mao, Yue Wang, Yifan Yang, Lu Li, Kexin Yuan, Huijun Cao, Zhilang Qiu, Guo Guo, Jianwei Wu, Jian Peng

**Affiliations:** a Key Laboratory of Infectious Immune and Antibody Engineering of Guizhou Province, Cellular Immunotherapy Engineering Research Center of Guizhou Province, School of Biology and Engineering/School of Basic Medical Sciences, Guizhou Medical University, Guiyang, China; b Key Laboratory of Environmental Pollution Monitoring and Disease Control, Ministry of Education, Guizhou Medical University, Guiyang, China; c The Key and Characteristic Laboratory of Modern Pathogen Biology, Basic Medical College, Guizhou Medical University, Guiyang, China; d Center for Clinical Laboratories, The Affiliated Hospital of Guizhou Medical University, Guiyang, China; Shenzhen Bay Laboratory

**Keywords:** *S. epidermidis*, antimicrobial peptide, antibacterial activity, antibacterial mechanism

## Abstract

Staphylococcus epidermidis is part of the normal microbiota that colonizes the skin and mucosal surfaces of human beings. Previous studies suggested that S. epidermidis possessed low virulence, but recent studies confirmed that it can acquire high virulence from Staphylococcus aureus and with the increasing detection of methicillin-resistant S. epidermidis. It has become a major pathogen of graft-associated and hospital-acquired infections. In previous studies, we modified the antimicrobial peptide Cec4 (41 amino acids) and obtained the derived peptide C9 (16 amino acids) showing better antimicrobial activity against S. epidermidis with an MIC value of 8 μg/mL. The peptide has rapid bactericidal activity without detectable high-level resistance, showing certain inhibition and eradication ability on S. epidermidis biofilms. The damage of cell membrane structures by C9 was observed by scanning emission microscopy (SEM) and transmission electron microscopy (TEM). In addition, C9 altered the S. epidermidis cell membrane permeability, depolarization levels, fluidity, and reactive oxygen species (ROS) accumulation and possessed the ability to bind genomic DNA. Analysis of the transcriptional profiles of C9-treated cells revealed changes in genes involved in cell wall and ribosome biosynthesis, membrane protein transport, oxidative stress, and DNA transcription regulation. At the same time, the median lethal dose of C9 in mice was more than 128 mg/kg, and the intraperitoneal administration of 64 mg/kg was less toxic to the liver and kidneys of mice. Furthermore, C9 also showed a certain therapeutic effect on the mouse bacteremia model. In conclusion, C9 may be a candidate drug against S. epidermidis, which has the potential to be further developed as an antibacterial therapeutic agent.

**IMPORTANCE** S. epidermidis is one of the most important pathogens of graft-related infection and hospital-acquired infection. The growing problem of antibiotic resistance, as well as the emergence of bacterial pathogenicity, highlights the need for antimicrobials with new modes of action. Antimicrobial peptides have been extensively studied over the past 30 years as ideal alternatives to antibiotics, and we report here that the derived peptide C9 is characterized by rapid bactericidal and antibiofilm activity, avoiding the development of resistance by acting on multiple nonspecific targets of the cell membrane or cell components. In addition, it has therapeutic potential against S. epidermidis infection *in vivo*. This study provides a rationale for the further development and application of C9 as an effective candidate antibiotic.

## INTRODUCTION

Staphylococcus epidermidis belongs to the coagulase-negative staphylococci, which are a normal bacterial community colonizing the skin and mucosal surfaces of the human body. However, when the skin or mucosal barrier is damaged, the immune function of the body is impaired, or the body receives implant-related treatment, it can invade the body or be transferred to other parts of the body by adhering to indent venous catheters, artificial heart valves, artificial joints, and other medical grafts, causing bacterial endocarditis, neonatal septicemia, and infection around the prosthesis. At present, it has become one of the most important pathogens of hospital-acquired infection (1, 2). Compared with the more virulent Staphylococcus aureus, S. epidermidis is less capable of secreting invasive enzymes and virulence factors, but it can avoid attacks of the host immune system and cause persistent infection (3). Recent studies have also found that S. epidermidis is also highly virulent. For example, TarIJLM is a genetic element in S. epidermidis which is closely related to invasiveness and virulence. The S. aureus type-wall teichoic acid (WTA) encoded by this gene enhances S. epidermidis adhesion and virulence, while the normal colonization ability in the nasal cavity was weakened, which led to their lifestyle changing from a symbiotic state to a pathogenic state (4). In addition, studies have shown that the pathogenicity and drug resistance of S. epidermidis are closely related to the formation of biofilm, and bacteria in the biofilm state are more resistant to antibiotics, external environmental pressure, and the host immune system, leading to the recurrence of infection (5, 6).

For a long time, antibiotics have played an important role in the prevention and treatment of infectious diseases. However, with the widespread use of antibiotics, such as broad-spectrum penicillin, aminoglycosides, and macrolides, the drug resistance rate of S. epidermidis has been increasing year by year and even the phenomenon of multiple drug resistance has appeared (7). Meanwhile, S. epidermidis is also considered a reservoir of drug resistance genes, and about 20% of its pan-genome is variable genome, which can accumulate different resistance genes and these resistance genes spread among Staphylococcus isolates (8). For example, the *mecA* gene on the *mec* (SCC*mec*) element of the staphylococcal cassette chromosome encodes a specific penicillin-binding protein (PBP2a), which endows bacteria with methicillin resistance (2). At present, vancomycin, as the preferred drug for the treatment of methicillin-resistant bacteria, has a good effect on systemic infection caused by Staphylococcus. However, its toxicity, low tissue permeability, drug resistance, and weak inhibition effect of biofilm have limited its clinical application (9, 10). The increasing problem of antibiotic resistance and the emergence of bacterial pathogenicity have prompted a series of studies on alternative drugs, highlighting the need for antimicrobial agents with new modes of action.

Antimicrobial peptides (AMPs) form a class of small-molecule polypeptides encoded by specific genes widely existing in organisms, with characteristics of a wide antimicrobial spectrum and low resistance to drugs. As an ideal substitute for antibiotics, AMPs have been extensively studied, and great progress has been made in the past 30 years (11). So far, the AMP database (APD3, https://aps.unmc.edu/home) has reported more than 3,324 antimicrobial peptides from bacteria, archaea, protists, fungi, plants, and animals. Currently, studies of the mechanism of action of AMPs are mainly focused on cell membranes, and it is generally believed that AMPs can bind to and interact with the negatively charged bacterial cell membranes, resulting in membrane potential disorder, membrane permeability change, and cell content leakage, which ultimately lead to cell death (12). In addition to causing severe damage to cell membranes, AMPs can also cross cell membranes and kill bacteria by interfering with normal cellular processes such as inhibiting the synthesis of DNA, RNA, protein, and cell wall (13). AMPs such as bacterial AMPs (14), the snake venom antimicrobial peptide pseudonajide (15), and the frog-derived antimicrobial peptide temporin-1CEC (16) have also been reported to exhibit better antibacterial and antibiofilm activity against S. epidermidis, further confirming that AMPs and their derivatives can be used as an effective way to develop novel antibacterial drugs.

In a previous study, we reported that a new member of the cecropin family, cecropin 4 (Cec4), has a high biosecurity profile and has shown good antibacterial activity and biofilm eradication against Acinetobacter baumannii (17, 18). Since the narrow antimicrobial spectrum of Cec4 and its long sequence increased the cost of synthesis, it has been modified to obtain a series of derived peptides with a broader antimicrobial spectrum and smaller molecular weight. Among them, C18 (16 amino acids) has been confirmed to have good antibacterial activity against methicillin-resistant Staphylococcus aureus (MRSA) and Candida albicans, which also has a certain therapeutic effect against infection *in vivo* (19, 20). Meanwhile, we also found that C9, which has only one amino acid substituted compared with C18, showed good antibacterial activity against S. epidermidis, with an MIC value of 8 μg/mL, which was more than 16 times higher than that of Cec4 (>128 μg/mL). In conclusion, this paper systematically studied the *in vitro* biological activity, antibiofilm ability, and antibacterial mechanism, as well as *in vivo* safety and efficacy of C9, expecting to provide theoretical and technical support for the development of new antibacterial drugs.

## RESULTS

### Antibacterial effect of C9 on S. epidermidis.

**FIG 1:**
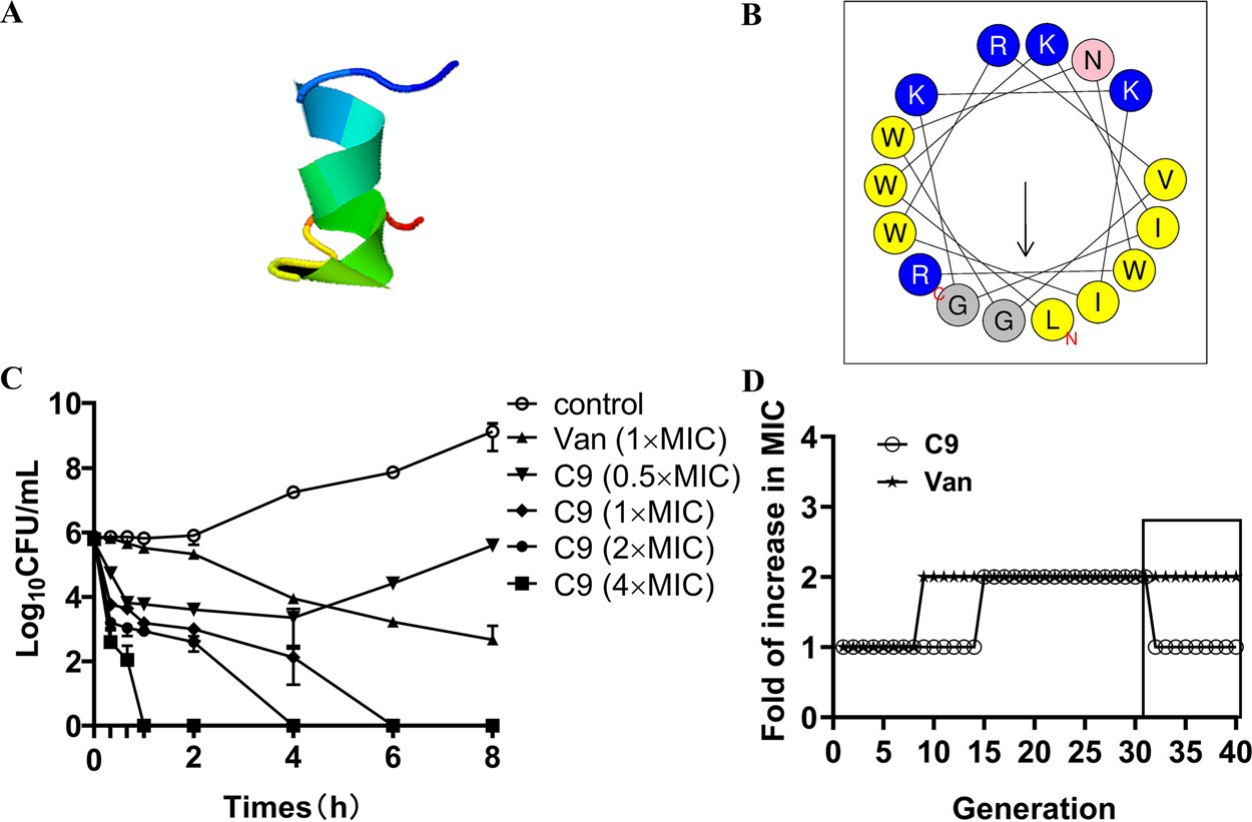
C9 shows bactericidal activity against S. epidermidis without detectable resistance development. (A) Three-dimensional structure projections of C9. (B) The helical wheel projection of C9; yellow circles denote hydrophobic amino acids, and blue circles denote hydrophilic amino acids. (C) Time-kill curve of C9 and vancomycin against S. epidermidis ATCC 35984; the experiments were conducted triplicate and presented as the mean ± SD. (D) Drug resistance development of S. epidermidis ATCC 35984 after subinhibitory doses of C9 and vancomycin (Van) treatment over 30 generations. The MIC changes of last 10 generations after passing the induced strain in the absence of the drug.

The three-dimensional structure prediction showed that C9 mainly presented an α-helix, and hydrophobic and hydrophilic amino acids were also found on both sides in the helical wheel prediction (Fig. 1A and B). In the antibacterial ability experiment, C9 exhibited great antibacterial activities against S. epidermidis ATCC 35984 and 34 clinical S. epidermidis strains (28 methicillin-resistant S. epidermidis [MRSE] strains and 6 methicillin-susceptible S. epidermidis [MSSE] strains), with a MIC of 8 μg/mL (see Table S1 in the supplemental material). The results of time-kill kinetics studies (Fig. 1C) showed that C9 exerts a rapid bactericidal efficacy in a dose-dependent manner, and the rate of killing was significantly faster than the killing kinetics of vancomycin. In the first 20 min, 1×, 2×, and 4× MIC of C9 reduced the number of colonies by 2.04 log_10_ CFU/mL, 2.59 log_10_ CFU/mL, and 3.19 log_10_ CFU/mL and killed all viable bacteria within 6 h, 4 h, and 1 h, respectively. However, the action of vancomycin even after 8 h of incubation achieved only a 3.17 log_10_ CFU/mL reduction in the viable bacterial count.

In *in vitro* resistance induction experiments, the MIC value of C9 against S. epidermidis was increased 2-fold after treatment with subinhibitory concentrations of C9 (1/2× MIC) for 15 passages, while vancomycin exhibited a 2-fold increase at the 9th passage (Fig. 1D). The C9-induced resistance disappeared, while the resistance induced by vancomycin still existed after subculturing without corresponding antibacterial drugs, indicating that S. epidermidis developed transient resistance under the condition of long-term use of C9. In combination with clinical antibiotics, the MIC of vancomycin (glycopeptide) and ciprofloxacin (fluoroquinolone) against S. epidermidis were reduced 2-fold; oxacillin (penicillin) MIC values for S. epidermidis 5 and S. epidermidis ATCC 35984 were reduced by 2-fold and 8-fold, respectively, with a fractional inhibitory concentration index (FICI) index between 0.625 and 1 (Table S2). Those results show that the combination of C9 and antibiotics has an additive effect. The antibacterial activity of C9 was reduced by 2-fold in the presence of physiological concentrations of Na^+^ and Ca^2+^, and its antibacterial activity was lost (MIC, >128 μg/mL) after being treated with trypsin. However, C9 activity has no significant change in 10% serum and K^+^. The above-described results show that C9 is not tolerant to trypsin but has certain stability in ions and serum (Table 1).

**TABLE 1:** The effects of salts, serum, and enzymes on C9 activity against S. epidermidis^*a*^

Strain	MIC (μg/mL) for:					
	C9	C9 + NaCl	C9 + KCl	C9 + CaCl_2_	C9 + serum	C9 + trypsin
S. epidermidis 5	8	16	8	16	8	>128
S. epidermidis ATCC 35984	8	16	8	16	8	>128

a The final concentrations of NaCl, KCl, CaCl_2_, serum, and trypsin were 150 mM, 4.5 mM, 2.5 mM, 10%, and 1 mg/mL, respectively.

### Effects of C9 on S. epidermidis biofilms.

**FIG 2:**
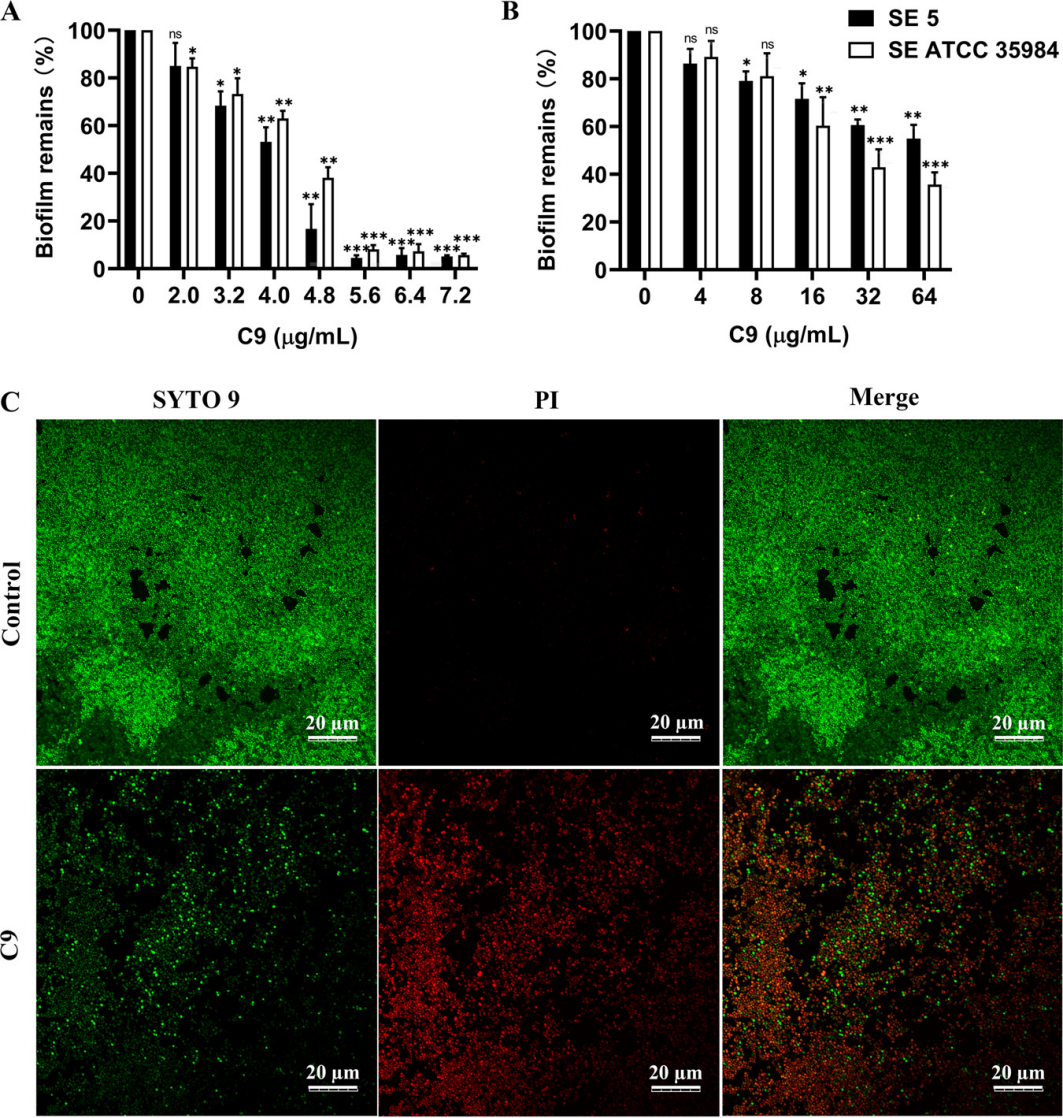
Effects of C9 on S. epidermidis biofilms. (A and B) Effects of C9 on biofilm formation (A) and established biofilm (B) of S. epidermidis ATCC 35984 and S. epidermidis 5. The adherent biofilm was stained with crystal violet and presented as the percentage of biofilm remaining in comparison with the untreated control (0× MIC). The statistics reflect the comparison between S. epidermidis 5 and S. epidermidis ATCC 35984 at different C9 concentrations with their respective controls (0 μg/mL). Results were presented as the mean ± SD. *, *P* < 0.05; **, *P* < 0.01; ***, *P* < 0.001. (C) Bactericidal effects of C9 (32 μg/mL) on mature biofilm of S. epidermidis ATCC 35984 observed in CLSM using SYTO 9 and PI staining. Bacterial cells stained with green fluorescence (SYTO 9) are viable, and those with red fluorescence (PI) are dead. Scale bar = 20 μm.

Biofilm inhibition and eradication experiments were performed on S. epidermidis 5 and S. epidermidis ATCC 35984 with strong biofilm-forming ability. C9 inhibited the biofilm formation of S. epidermidis in a dose-dependent manner (Fig. 2A), with a more than 26.7% or 31.64% reduction in S. epidermidis ATCC 35984 or S. epidermidis 5 biofilm formation after 24 h of treatment with C9 (3.2 μg/mL). When the C9 concentration reached 0.7× MIC (5.6 μg/mL), the biofilm formation of the two strains could be both reduced by 90% (*P < *0.001). In addition, the amount of mature biofilm was significantly reduced after treatment with C9 (Fig. 2B). The decrease of mature biofilms of S. epidermidis ATCC 35984 and S. epidermidis 5 at 8× MIC (64 μg/mL) can reach about 45 to 64%. The confocal laser scanning microscopy (CLSM) images also showed that the intensity of green fluorescence signal was significantly reduced after C9 treatment, most bacteria were stained red, and the distribution of bacteria was relatively sparse (Fig. 2C). It is suggested that C9 can inhibit the formation of biofilm and has certain destructive effects on mature biofilm.

### Antibacterial mechanism of C9 against S. epidermidis.

**FIG 3:**
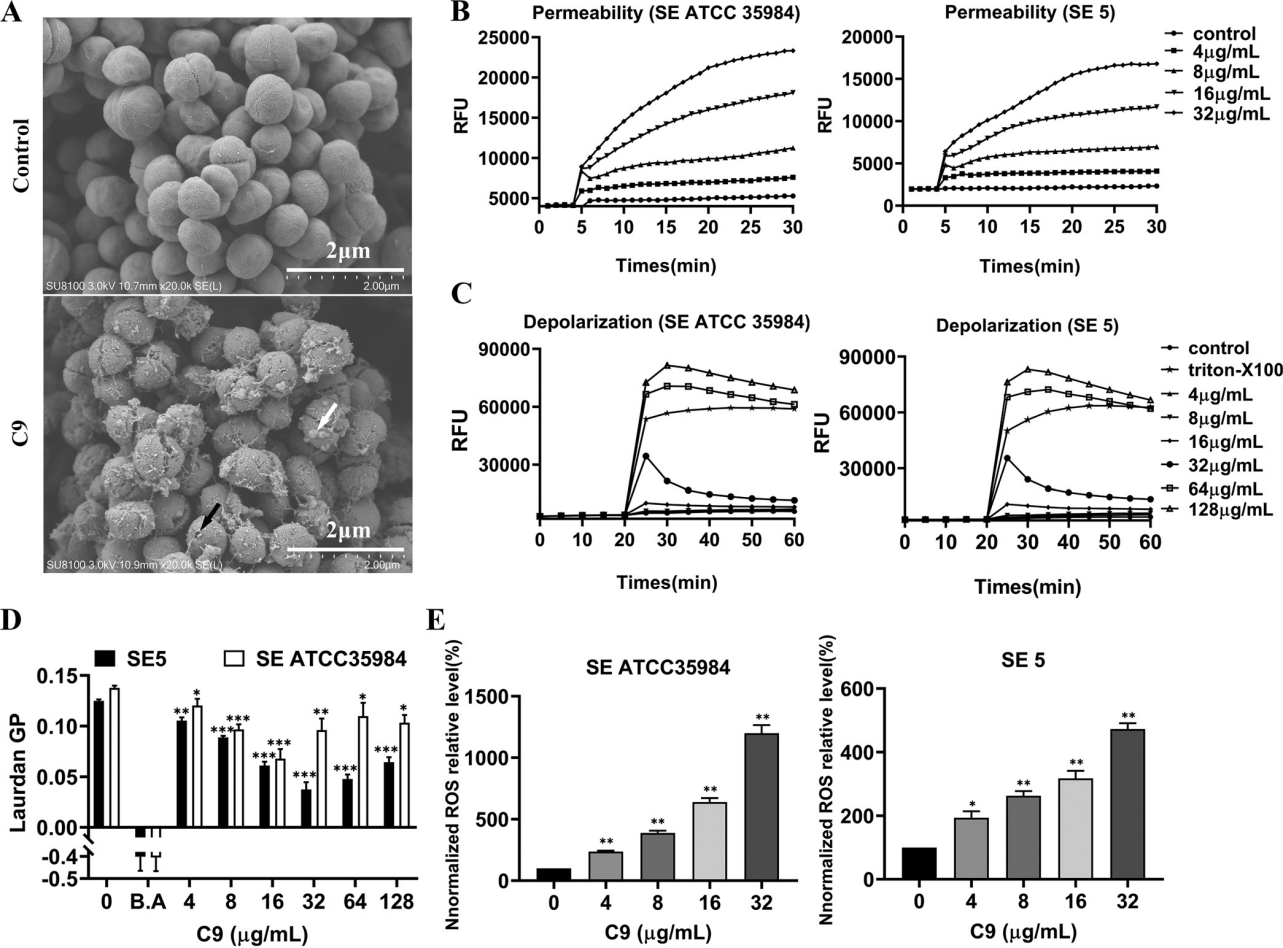
Membrane active mechanism of actions by C9. (A) Scanning electron microscopy (SEM) images of S. epidermidis ATCC 35984 treated with C9 (2× MIC) for 2 h. Black arrows represent perforations and white arrows represent bubbles or small protrusions. The scale bar is 2 μm. (B and C) The dynamic curves of the membrane-impermeable fluorescent dye PI (B) and the membrane potential-sensitive dye DISC3-5 (C) reflect the changes of membrane permeability and membrane depolarization after C9 treatment of S. epidermidis. PBS was used as a negative control. Individual data points (*n* = 3) and means are shown; error bars not shown for clarity. (D) Membrane fluidity of S. epidermidis ATCC 35984 (SE ATCC 35984) and S. epidermidis 5 (SE 5) after treatment with C9 was evaluated based on Laurdan generalized polarization (Laurdan GP). Benzyl alcohol (B.A) served as the control for fluidization. (E) Total ROS accumulation in S. epidermidis ATCC 35984 and S. epidermidis 5 was determined by DCFH-DA; before the fluorescence assay, probe-labeled cells were treated with C9 at 37°C for 1 h. Results are presented as the mean ± SD. *, *P* < 0.05; **, *P* < 0.01; ***, *P* < 0.001.

To elucidate the antibacterial mechanism of C9 against S. epidermidis, the effect of C9 on bacterial morphology and structure was first observed under SEM, and the untreated S. epidermidis had normal morphology and a smooth surface (Fig. 3A). However, after C9 treatment, the structure became rough, and its integrity was destroyed, with perforations (black arrow), bubbles (white arrow), and filamentous adhesions observed. Propidium iodide (PI) was subsequently used to detect changes in membrane permeability, and the dye was able to enter cells through broken cell membranes and bind to bacterial DNA, resulting in increased fluorescence intensity. The results showed that 4 μg/mL C9 caused a significant increase in fluorescence intensity of S. epidermidis ATCC 35984 and S. epidermidis 5 and showed a dose- and time-dependent manner (Fig. 3B), which further confirmed that C9 can disrupt the integrity of the S. epidermidis cell membrane.

In addition, the membrane potential-sensitive probe DISC3-5 can be inserted into polarized membranes and released when the membrane is depolarized, causing the fluorescence signal to increase. The results showed that C9 dissipated the membrane potential of S. epidermidis ATCC 35984 and S. epidermidis 5 in a dose-dependent manner, with concentrations of 64 μg/mL, comparable to the level of depolarization induced by 1% Triton X-100 (Fig. 3C). In addition, the fluorescence polarizing probe Laurdan’s (6-dodecanoyl-*N*,*N*-dimethyl-2-naphthylamine) detection of membrane fluidity is based on a fluorescence emission shift that depends on the number of water molecules surrounding the probe and reflects lipid head group diffusion and fatty acid chain fluidity, quantified by Laurdan generalized polarization (GP), which ranges from −1 (flow and disorder) to +1 (rigid and ordered). The results showed that, like the membrane fluidizing agent benzyl alcohol (BA), the GP values decreased after C9 treatment and showed a dose-dependent effect at concentrations of 16 μg/mL (S. epidermidis ATCC35984) or 32 μg/mL (S. epidermidis 5) (Fig. 3D). Furthermore, the accumulation of reactive oxygen species (ROS) can be reflected by the fluorescence intensity of oxidized 2,7-dichlorodihydrofluorescein (DCFH). DCFH diacetate (DCFH-DA) can penetrate the bacterial cell membrane and be hydrolyzed to produce DCFH, which is oxidized to produce green fluorescence. The results showed that C9 triggered the production of reactive oxygen species in S. epidermidis in a concentration-dependent manner (Fig. 3E).

**FIG 4:**
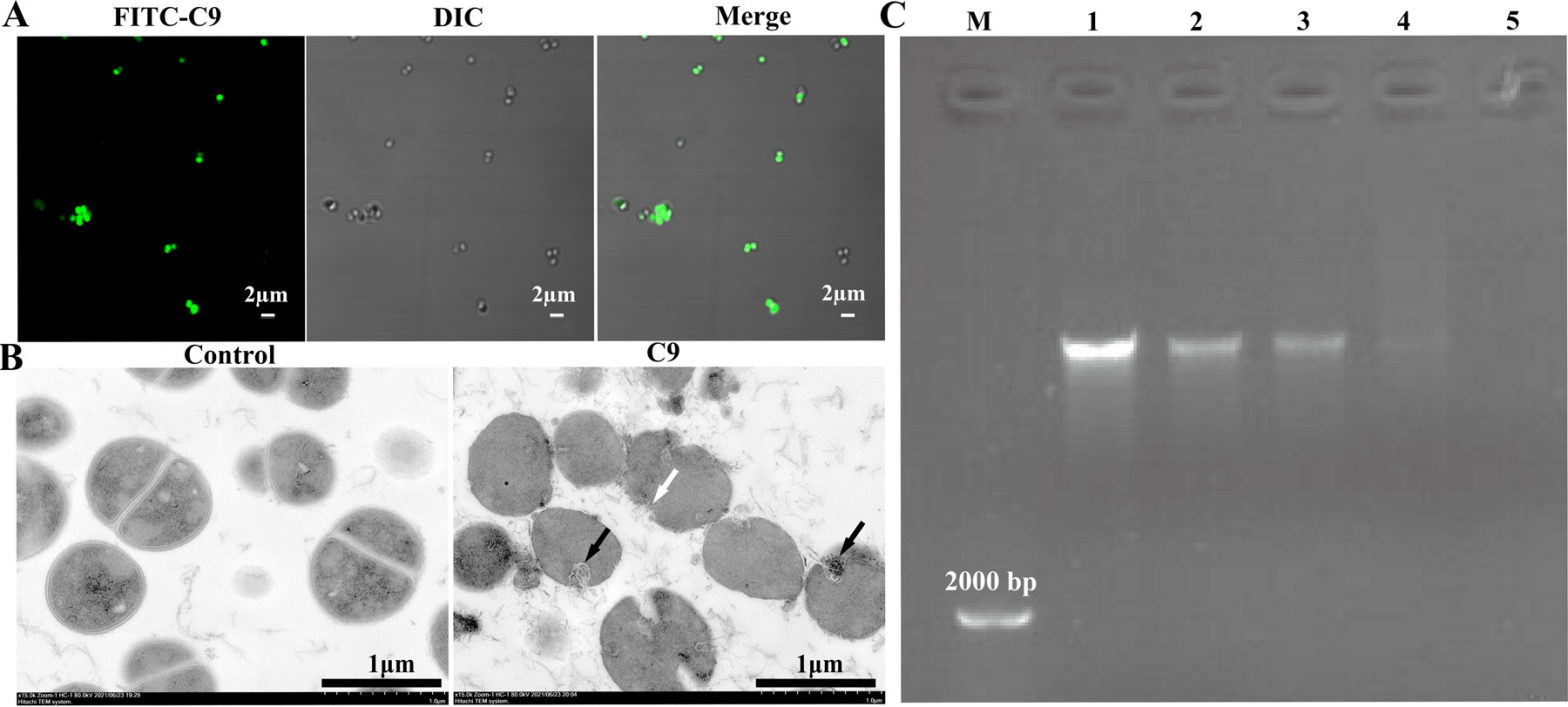
Interaction of C9 with intracellular material. (A) CLSM shows the localization of C9 in S. epidermidis ATCC 35984 after treatment with FITC-C9 at 1× MIC for 1 h. Scale bar = 2 μm. (B) Transmission electron microscopy (TEM) images of S. epidermidis ATCC 35984 treated with C9 (2× MIC) for 2 h. Scale bar = 1 μm. (C) Gel retardation analysis of the binding of C9 to S. epidermidis ATCC 35984 DNA. Lane M, DL2000 DNA marker; lanes 1 to 5, the genomic DNA treated with C9 at 0, 8, 16, 32, and 64 μg/mL, respectively, at 37°C for 1 h.

CLSM showed that most of the bacteria showed green fluorescence after 1× MIC FITC-C9 treatment (Fig. 4A), indicating that C9 may enter the cell through the damaged cell membrane. The morphological and structural changes of S. epidermidis treated with C9 was further observed by TEM. The untreated cells showed clearly visible structures, with intact cell membrane and wall. In contrast, most of the bacteria showed apparent structural alteration after C9 (2× MIC) treatment for 1 h (Fig. 4B), including disappearance of the cell wall, disruption of the cell membrane with blurred borders, and clustering of the intracellular contents (black arrows). It has been reported that some antimicrobial peptides may have membrane attack activity and nucleic acid binding effect. Since DNA carries a large negative charge, positively charged AMPs can bind to the phosphate of DNA through electrostatic interactions, resulting in delayed DNA migration (21). To test the DNA binding ability of C9, different concentrations of C9 were mixed with S. epidermidis genomic DNA. The result of agarose gel retardation showed that the electrophoretic migration of DNA decreased progressively with increasing C9 concentration, and the bands after 32 μg/mL treatment were scattered in the lane, indicating that C9 can bind to the genomic DNA of S. epidermidis (Fig. 4C).

**FIG 5:**
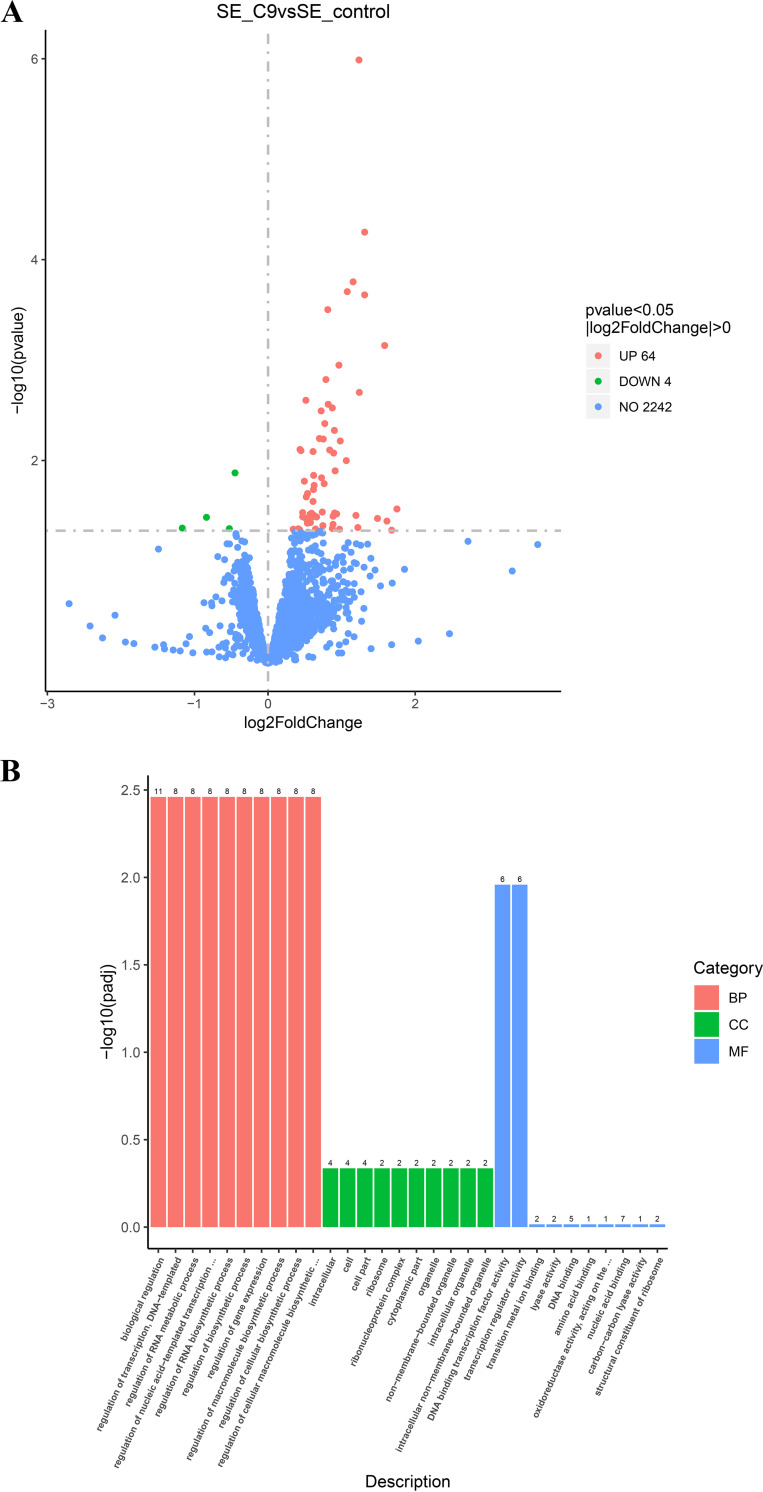
Transcriptome analysis of S. epidermidis ATCC 35984 after exposure to C9. (A) Volcano plot annotation analysis of the differential expression genes (DEGs) in S. epidermidis ATCC 35984 after exposure to C9 (4 μg/mL) for 3 h. Significantly differentially expressed genes were treated with red dots (upregulated) or green dots (downregulated). The abscissa represents the fold change, and the ordinate represents the statistical significance. (B) Gene Ontology (GO) annotation analysis of the DEGs in S. epidermidis ATCC 35984, broadly separated into biological processes (BP), cellular components (CC), and molecular function (MF).

Some studies have shown that in antibiotic therapy, due to the influence of drug pharmacokinetics and drug diffusion barriers, pathogens can often resist the killing of antibiotics by producing a series of stress effects under the subinhibitory concentration of antibiotics (22, 23). To understand the molecular mechanism of S. epidermidis underlying the action of subinhibitory C9 and the gene expression changes at the mRNA level, transcriptional analysis revealed 68 differentially expressed genes, 64 of which were upregulated and 4 of which downregulated (Fig. 5A). Gene Ontology (GO) annotation analysis indicated that the upregulated differentially expressed genes were associated with biological processes (e.g., regulation of biosynthetic processes), cellular components (e.g., ribosomes), and molecular functions (e.g., catalytic activity and binding) (Fig. 5B). Specifically, three genes (*dltD*, *vraX*, and RS05660) were associated with cell wall synthesis, three genes (RS02955, RS02960, and RS01850) were responsible for membrane transporters, three genes were responsible for electron transport (RS02315, RS02195, and RS08715), five genes belonged to the ribosomal protein family (including *rpsO*, *rpsJ*, and RS11120), three genes were related to oxidative stress (*tpx*, RS09670, and RS11790) and two genes were related to amino acid biosynthesis (*argF* and RS05645). Meanwhile, we found that the DNA binding transcription factor activity (GO:0003700: DNA binding transcription factor activity) and transcription regulator activity (GO:0140110: transcription regulator activity) pathways were jointly enriched for the same six genes (RS06770, RS03200, RS02455, RS02830, RS10285, *ccpA*), which are all transcriptional regulatory repressors (Table S3). To further confirm the RNA sequencing (RNA-Seq) results, a total of seven genes (five upregulated and two downregulated) associated with cell wall, cell membrane, or ribosome were selected for quantitative rea-time PCR (qRT-PCR) analysis. The qRT-PCR results showed that the expression levels of the assayed genes have a consistent change with the RNA-Seq data, indicating that the RNA-Seq data were reliable (Fig. S1).

**FIG 6:**
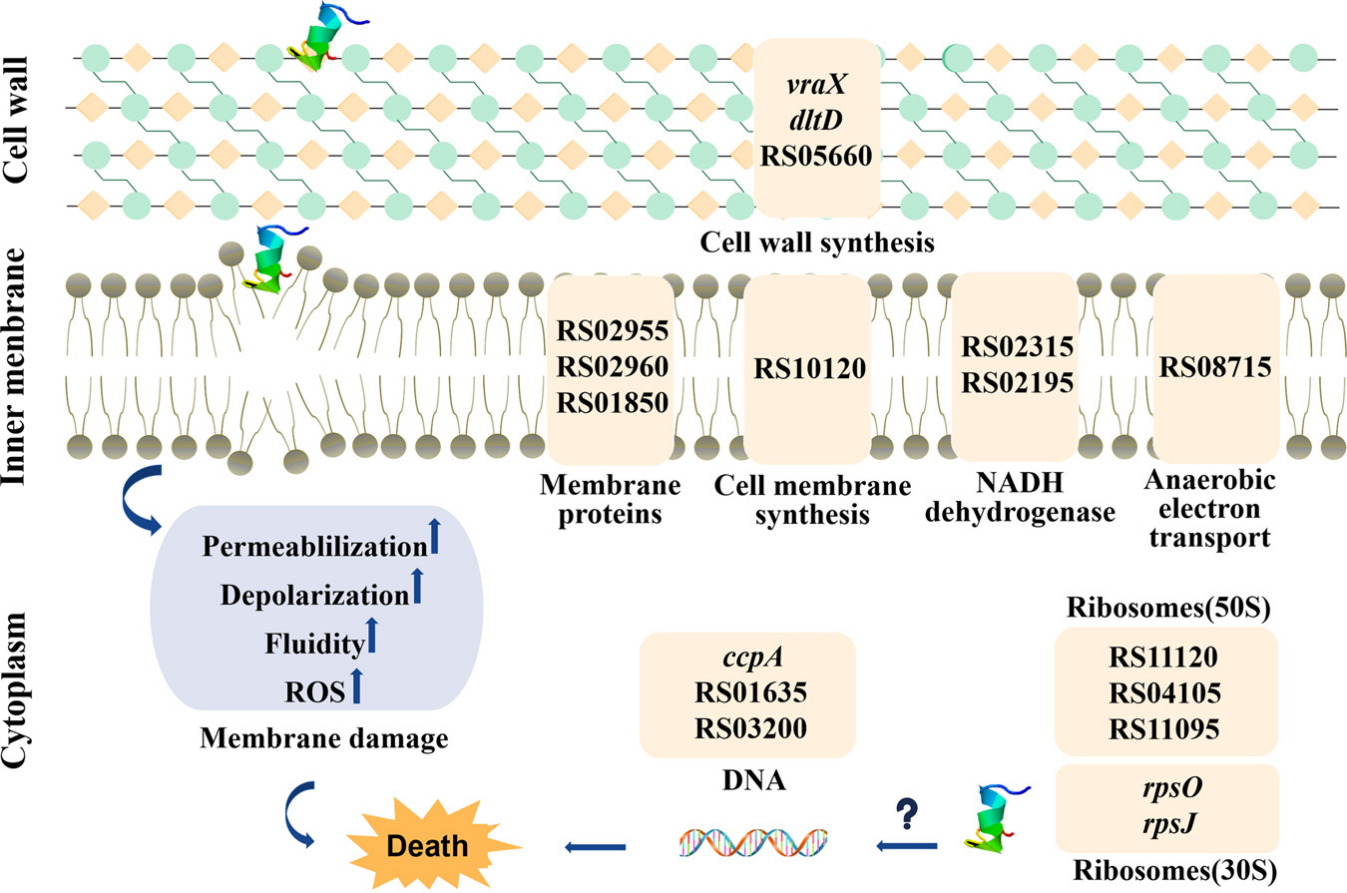
Schematic representation for the mechanism of action of C9 against S. epidermidis. It is possible that C9 kills the S. epidermidis by crossing the cell wall and destroying cell membrane integrity, further resulting in membrane dysfunction, or acting on intracellular targets, to affect the transcriptional regulation of DNA and ribosomal biosynthesis, which in turn affects the normal physiological activities of bacteria. Only the DEGs relevant to this study are shown in Table S3. Some genes do not have corresponding gene names assigned. In these cases, “RS05660” at the top of the figure corresponds to gene ID EQW00_RS05660.

In a brief, the schematic representation for the mechanism of action of C9 against S. epidermidis is shown in Fig. 6. C9 can penetrate the cell wall of S. epidermidis, thereby destroying the integrity of the cell membrane, resulting in changes to membrane permeability, fluidity, and potential and triggering the accumulation of ROS. In addition, it can also enter the cell, affecting the transcriptional regulation of DNA and biosynthesis of ribosomes, thus affecting the normal physiological activities of bacteria.

### Safety and efficacy of C9.

**FIG 7:**
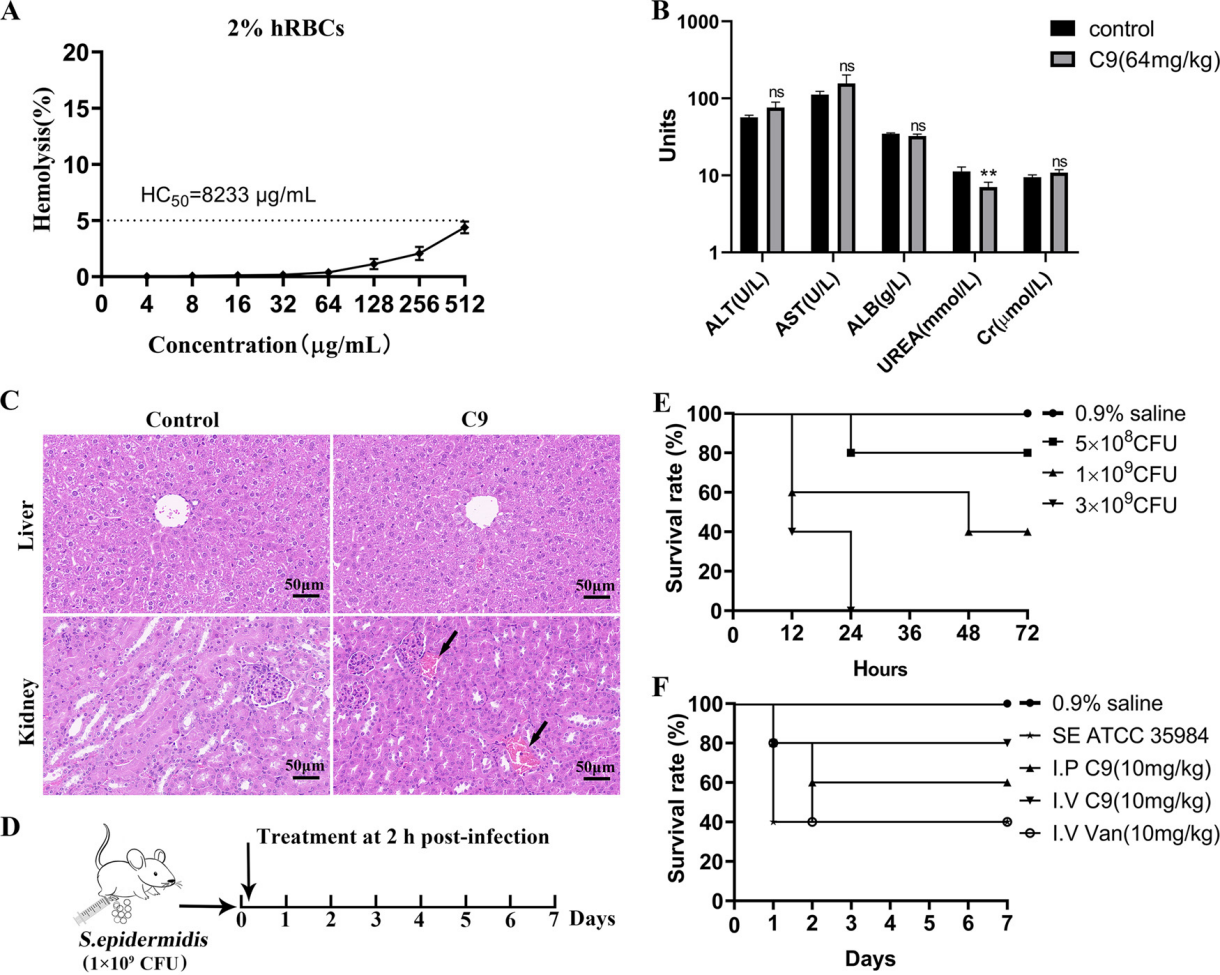
Toxicity assessment of C9 and the therapeutic efficacy on mouse bacteremia. (A) Hemolysis of C9 against human red blood cells. Triton X-100 (0.1%) and PBS were used as positive and negative controls, respectively. HC_50_ is the concentration of the peptides that resulted in 50% lysis of red blood cells. (B) Blood biochemical assays of mice for 24 h of C9 treatment (64 mg/kg). Serum levels of alanine aminotransferase (ALT), aspartate aminotransferase (AST), albumin (ALB), urea nitrogen (UREA), and blood creatinine (Cr) were determined. Data are presented as means ± SD (5 mice per group), and the statistical analysis was assayed by the independent samples *t* test (C) Histopathology observations of the liver and kidney after C9 (64 mg/kg) treatment, kidney venous congestion is indicated by a black arrow. (D) Scheme of the experimental protocol for the mouse bacteremia model. (E) The survival rate changes after infection with S. epidermidis (5 mice per group). (F) The survival rate changes after C9 and vancomycin treatment (5 mice per group).

To assess the potential value of C9 in clinical application, the toxic effect of C9 on erythrocytes was first examined *in vitro*. It was found that C9 displayed no substantial hemolysis at all concentrations tested (hemolysis rate, < 5%), and the concentration of the peptides that resulted in 50% lysis of red blood cells (HC_50_) was 8,233 μg/mL (Fig. 7A), indicating that C9 had no hemolytic effect. The acute toxicity of C9 in mice was further evaluated, and blood biochemical analysis and histopathology were performed in mice inoculated with C9 (64 mg/kg) or normal saline. The results showed that all mice survived after intraperitoneal injection of C9 (32 to 128 mg/kg) (data not shown), with a median lethal dose (LD_50_) of >128 mg/kg, but the body weight gain of mice was affected when the concentration reached 128 mg/kg (Fig. S2). The blood urea level of mice was significantly decreased after 24 h of intraperitoneal administration of C9 (64 mg/kg) (Fig. 7B), and venous blood stasis (Fig. 7C) was found in kidney tissue. There was no significant difference in serum levels of ALT, AST, ALB, Cr, and liver tissue between the two groups (*P > *0.05), suggesting that the higher dose of C9 caused slight toxicity to the kidneys.

To further explore the therapeutic effect of C9 in the mouse model, a level of 60% mortality (1 × 10^9^ CFU) was selected for infection by intraperitoneal injection (Fig. 7D and E) and treatment with C9 or vancomycin by intraperitoneal or intravenous administration 2 h later. The survival curve showed that the survival rate of the model group was 40% and the 7-day survival rate of the mice was 60% and 80%, respectively, when treated by intraperitoneal or tail vein injection of C9 (10 mg/kg) (Fig. 7F).

## DISCUSSION

Antibiotic resistance (AMR) has become a global crisis and poses a major threat to public health. Antibiotic-resistant infections cause 700,000 deaths worldwide every year, and this number is expected to rise to 10 million annually in 2050 (24). In contrast to the single target of traditional antibiotics, AMPs can be highly active against resistant bacteria by acting on multiple nonspecific targets in cell membranes or cells, avoiding the development of drug resistance (25).

In this study, the derived peptide C9, which was modified from the housefly cecropin Cec4, had good antibacterial activity against S. epidermidis (including MSSE and MRSE), with an MIC of 8 μg/mL, being able to rapidly exert a bactericidal effect in a short time (20 min), while vancomycin usually takes a longer time to achieve the bactericidal purpose (26). Like the induction of Pseudomonas aeruginosa by defensin LL-37 (27), C9 also produced transient resistance following the *in vitro* induction of S. epidermidis, while vancomycin-induced resistance persisted. The activity of antimicrobial peptides is easily affected by the humoral environment, such as ions, serum, and proteases (28). Among them, Na^+^ and Ca^2+^ have been reported to decrease the activity of peptides by affecting the electrostatic interaction between the peptide and bacteria and may prevent peptides from entering the bacterial membrane and result in decreased AMP antimicrobial activity (29, 30). In this study, C9 activity was also slightly affected in the presence of Na^+^ and Ca^2+^. However, the loss of C9 activity after trypsin treatment was probably due to the C terminus of lysine (K, 3) and arginine (R, 2) in the C9 sequence degradation by trypsin (31). In addition, the binding of albumin to AMP in serum also resulted in a decrease of antimicrobial activity (32), while C9 activity in 10% serum was not affected. The above described results indicate that C9, when used as a rapid bactericide, does not produce high levels of resistance and maintains a certain degree of stability.

Biofilm is a three-dimensional community of microorganisms embedded in an extracellular polymeric matrix (EPS). EPS is composed of polysaccharides, extracellular DNA (eDNA), teichoic acid, and proteins (6) and acts as a physical barrier for the penetration of drugs, which reduces the susceptibility of bacteria to antimicrobial agents and leads to the recurrence of infection (33). Therefore, preventing or inhibiting the formation of biofilms is particularly critical. However, subinhibitory concentrations of antibiotics (tigecycline, rifampicin, and vancomycin) have been reported to promote the formation of staphylococcal biofilms (10, 34). It was found that C9 obviously prevented static biofilm formation at 0.4 to 0.8× MIC and eradicated the preformed biofilm of S. epidermidis at higher concentration, and CLSM images further illustrated the killing effect of C9 on static biofilm bacteria. Altogether, the above-described results indicated that C9 is not only antibacterial but is also a potent antibiofilm agent.

As an important place for bacterial respiration, cell wall synthesis, and energy metabolism, the cell membrane also controls the transport of secreted proteins and myriad small molecules between the intracellular and extracellular space, and its integrity is a key factor in the barrier function of the cell membrane (35). Previous studies have shown that the compounds 3-p-trans-coumaroyl-2-hydroxyquinic acid (CHQA) and bithionol can damage the cytoplasmic membrane of S. aureus, mainly causing significant membrane depolarization with a loss of membrane integrity or increasing membrane fluidity, and result in passive permeabilization, leakage of cellular components, and bacterial death (36, 37). In this study, SEM and TEM showed the disruption of the bacteria by C9. Then, the increase in membrane permeability, depolarization level, and membrane fluidity were detected by PI, DISC3-5, and Laurdan dyes, respectively, and the changes of membrane permeability and fluidity occurred at lower concentrations (0.5× MIC), while the obvious changes of membrane potential required higher concentrations of C9 (8× MIC), further confirming the damage of C9 to the membrane of S. epidermidis. In addition, C9 also triggers the accumulation of total cellular ROS, which can damage DNA, RNA, proteins, and lipids when levels exceed the detoxification and repair capabilities of organisms, leading to bacterial cell death and a key factor in antimicrobial-mediated killing (38, 39). These data suggest that bacterial cell membranes are potential antibacterial targets for C9.

Transcriptome analysis showed a total of 64 upregulated differential expression genes (DEGs). Specifically, cell wall-related genes in bacteria upregulated under C9 treatment, among them, RS05660 and *dltD*, are involved in the biosynthesis of peptidoglycan in the cell wall (40) and the esterification of lipoteichoic acid by d-alanine (41), thereby affecting the biosynthesis of the bacterial cell wall. While *vraX* is upregulated after exposure to drugs related to cell membrane or cell wall metabolic damage, such as penfluridol (42) and vancomycin (43). In addition, RS02830 is a MarR family transcriptional regulator with a homolog protein in S. epidermidis, TcaR, which is involved in staphylococcal resistance to teicoplanin and methicillin (44); this regulator also acts as a negative transcriptional regulator of the *ica* locus and is involved in the regulation of proteins related to the biosynthesis of poly-β-1,6-*N*-acetyl-d-glucosamine (PNAG; negative regulation) (45). RS10285 is a DeoR/GlpR transcriptional regulator, possibly with sequence homology to LacR in S. aureus (46). In Staphylococcus, TcaR and LacR are weak and strong negative regulators, respectively, of the *ica* locus (45), and their high expression indicates additional repression of the *ica* locus, which may impair cell wall formation and alter cell permeability. RS03200 is a MurR**/**RpiR family of transcriptional regulators that repress MurQ transcription by binding to the MurQ promoter, affecting bacterial cell wall formation and intracellular metabolism of peptidoglycan to some extent (47). RS02455 is a GntR family transcriptional regulator, and the GntR-like proteins have membrane-associated transporter protein regulatory activity in Mycobacterium tuberculosis (48). Therefore, the GntR family in S. epidermidis may be involved in regulating the transcription of genes related to membrane transport proteins, causing upregulation of membrane-associated genes. The diacylglycerol kinase encoded by RS10120 could catalyze the phosphorylation of diacylglycerol to phosphatidic acid and promoted the biosynthesis of cell membrane components (49), with cytochrome ubiquinone oxidase (RS08715) and C4-dicarboxylate transporter (RS01850) mainly involved in bacterial respiration (50).

Oxidative stress-related genes were also upregulated in expression after C9 treatment. RS05650 is catabolic control protein A (*ccpA*), which showed upregulation in the transcriptome after C9 treatment. The protein-protein Interaction (PPI) results suggest a possible indirect regulatory relationship between RS10285 and this gene, and the upregulation of this gene may be a result of the secondary stress response of bacterial cells to C9. On the other hand, MarR family transcriptional regulators repress the transcription of *marA* genes in Escherichia coli, which leads to resistance to oxidative killing and enhances the survival of E. coli in an oxidative environment (51). Thus, with the rise in intracellular ROS due to C9, it becomes more likely to lead to bacterial cell death. In addition, the gene RS09745, encoding the phenol-soluble regulatory protein PSMε, was downregulated, suggesting that C9 may reduce the expression of virulence factors and biofilm formation in S. epidermidis (52). Genes belonging to bacterial metabolic pathways were also found to be upregulated, mainly including transcription factors associated with DNA binding, ribosomal protein components, translation, and glycolysis-related genes. In conclusion, C9 may cause upregulation of transcriptional regulatory repressors, which leads to alterations in many normal intracellular physiological metabolisms, and affect the normal physiological activity of bacteria by interfering with the biosynthesis of bacterial cell walls and ribosomes and the function of membrane proteins.

The safety and *in vivo* efficacy of a drug is one of the key factors determining whether a drug has potential research and development value, and mice are commonly used as mammalian models to assess the toxicity and efficacy of new antibacterial agents *in vivo* (53). This study evaluated the *in vivo* efficacy of C9 in a mouse model of peritoneal infection and found that a single caudal vein and intraperitoneal administration showed a therapeutic potential. As an *in vivo* therapeutic agent, it is particularly important to evaluate its acute toxicity in mammals. Intraperitoneal injection of C9 (32 to 128 mg/kg body weight) did not cause death in mice, and the dose of 64 mg/kg only slightly affected the renal tissue. No significant histological pathological changes were observed, indicating that C9 is relatively safe even for long-term use.

In conclusion, C9 has the characteristics of rapid bactericidal and antibiofilm activity without strong drug resistance. It can exert its antibacterial effect by destroying the cell membrane structure, changing the cell membrane permeability, depolarization level, and cell membrane fluidity, and triggering ROS accumulation. At the same time, it can also affect the biosynthesis of the cell wall/ribosome, the function of membrane proteins, and the regulation of DNA transcription, thus interfering with the normal biological function of bacteria. In addition, C9 has lower toxicity and therapeutic potential against S. epidermidis infection *in vivo*. The findings presented here may provide a great rationale for the further development and application of C9 as a potent antibiotic candidate.

## MATERIALS AND METHODS

### Bacterial strains, antimicrobial peptides, and reagents.

Clinical S. epidermidis isolates were collected from a tertiary hospital in Guiyang, China, and S. epidermidis ATCC 35984 was purchased from Beijing Baiobowei Biotechnology Co., Ltd. S. epidermidis was cultivated overnight at 37°C and 220 rpm in tryptone soy broth (TSB) and then diluted at 1:100 and incubated until the logarithmic phase for experiments. C9 was synthesized by solid-phase chemistry by Gil Biochemical Co., Ltd. (Shanghai, China) with purity of >95% and stored at **−**20°C, the amino acid sequence and physicochemical properties of the antimicrobial peptides are shown in Table S4. All reagents and chemicals were obtained from Solarbio Life Sciences (Beijing, China) unless otherwise stated.

### Structure analysis of C9.

The three-dimensional structure of C9 was predicted using the I-TASSER structure prediction server (https://zhanggroup.org/I-TASSER/). The helical wheel analysis was performed using the web program HeliQuest (https://heliquest.ipmc.cnrs.fr).

### MIC.

The MIC values of C9 and other traditional antibiotics were determined by a broth microdilution method according to the recommendations of the Clinical and Laboratory Standards Institute (CLSI) (54). Briefly, 100 μL C9 or other antibiotic was 2-fold serially diluted in Mueller-Hinton broth (MHB) and mixed with an equal volume of bacterial suspension (1 × 10^6^ CFU/mL) into a 96-well plate (NEST Biotechnology, China). The MIC was determined to be the lowest concentration of the peptide that resulted in no bacterial growth after incubation at 37°C for 18 to 20 h.

### Stability test.

To investigate the effect of salts, serum, and trypsin on the antibacterial activity of C9(29), the MIC was again tested as described in the previous section following the addition of NaCl (150 mM), KCl (4.5 mM), CaCl_2_ (2.5 mM), and serum (10%; Tianhang Biotechnology, China) to MHB. In addition, C9 was incubated with trypsin (1 mg/mL) for 1 h at 37°C, and the enzymatic activity was eliminated at 60°C before MIC determination.

### Time-kill kinetics.

The time-kill kinetics of C9 against S. epidermidis ATCC 35984 was measured following a previously described method (55). An equal volume of bacterial suspension (1 × 10^6^ CFU/mL) was mixed with C9 (0 to 32 μg/mL) or vancomycin (2 μg/mL). Samples were taken at 0, 20, 40, 60, 120, 240, 360, and 480 min, diluted, and plated onto Trypticase soy agar (TSA) agar plates. The number of viable colonies was determined by plate counts after incubation at 37°C for 24 h.

### Checkerboard assays.

The combined antimicrobial action of C9 and other antibiotics was assessed using the checkerboard assay as described previously (42). Bacterial suspensions (1 × 10^6^ CFU/mL) were added into 96-well plates (100 μL/well), and then a series of concentrations (0.125 to 8× MIC) of C9 and antibiotics (vancomycin, ciprofloxacin, and oxacillin) was added. MICs were recorded after 18 to 24 h of incubation at 37°C. The fractional inhibitory concentration index (FICI) was calculated: FICI = MIC_A_ (combination)/MIC_A_ (alone) + MIC_B_ (combination)/MIC_B_ (alone). The minimal value of the FICI was judged as follows: FICI ≤ 0.5, synergy; 0.5 < FICI ≤ 4, addition; FICI > 4, antagonism.

### Drug resistance.

Drug resistance of S. epidermidis induced by C9 was detected according to previous research methods (56). For this experiment, the MIC of C9 and vancomycin against S. epidermidis ATCC 35984 were determined, and the bacterial suspension exposed to 0.5× MIC was resuspended and grown to the logarithmic phase to determine the MIC with corresponding drugs. The above-described steps were repeated to 30 generations, and then the 30^th^-generation strain was subcultured without drugs and the MIC were recorded.

### Biofilm inhibition assay.

The effect of C9 on the biofilm formation of S. epidermidis ATCC 35984 and S. epidermidis 5 was assessed using the crystal violet (CV) method (57). Briefly, the bacterial suspension (1 × 10^6^ CFU/mL) was prepared in Trypticase soy broth (TSB) medium (supplemented with 2.5% glucose), and 100 μL was distributed to 96-well plates; then 100 μL C9 (0 to 7.2 μg/mL) was added and cultured for 24 h. The wells were gently washed twice with phosphate-buffered saline (1× PBS, 0.01 M, pH 7.2 to 7.4) to remove nonadherent cells and fixed with 200 μL methanol for 15 min. Then the well was stained with 200 μL 0.1% CV for 20 min and washed with 1× PBS. Finally, 200 μL 95% ethanol was added to all wells to dissolve the crystal violet stain and measured at 570 nm with a microplate spectrophotometer (Bio Tek, USA), and the biofilm remaining ratio was calculated.

### Biofilm eradication assay.

In brief, the mature biofilms were constructed with 200 μL bacterial suspension (1 × 10^6^ CFU/mL) cultured for 48 h and treated with C9 (0 to 32 μg/mL) for 24 h. The biofilm was quantitatively analyzed according to the above-described method, and the remaining ratio of biofilm was calculated.

### Confocal laser scanning microscopy (CLSM) of biofilms.

CLSM analysis was carried out according to a previous study (18). The S. epidermidis ATCC 35984 (1 × 10^6^ CFU/mL) was added to a 6-well plate (NEST Biotechnology, China) containing poly-lysine-treated coverslips and incubated for 48 h to form a mature biofilm. Then, the samples were treated with C9 (32 μg/mL) and TSB medium for 2 h. The biofilm staining was performed with SYTO 9 (5 μM; Invitrogen, USA) and PI (5 μM; Sigma, USA) for 15 min, and images were captured with CLSM (FV1000 microscope, Olympus, Japan).

### Scanning and transmission electron microscopy (SEM and TEM).

Morphological changes in S. epidermidis ATCC 35984 were observed according to previous reports with some modifications (56). The S. epidermidis cells (1 × 10^8^ CFU/mL) were treated with C9 (2× MIC) for 2 h at 37°C before being fixed in 2.5% glutaraldehyde at 4°C overnight. The samples were dehydrated by ethanol, dried in a critical point dryer, sputter-coated with gold, and observed with SEM (SU8100; Hitachi, Japan). Additionally, the samples were fixed with 1% OsO_4_, dehydrated by ethanol, embedded in resin, ultrathin-sectioned, stained with uranium acetate and lead citrate, and then observed using a TEM (HT7800; Hitachi, Japan).

### Membrane permeability assay.

Briefly, the S. epidermidis ATCC 35984 and S. epidermidis 5 were resuspended in PBS to 1 × 10^8^ CFU/mL, and 190 μL of cells containing PI (10 μM) was distributed in a black microplate (Corning, USA). The fluorescence intensity was detected using a Synergy H4 multifunctional microplate reader (Bio Tek, USA) at the 535 nm excitation and 610 nm emission wavelength for 4 min. Subsequently, 10 μL of C9 (0 to 32 μg/mL) was added and monitored continuously for another 25 min (58).

### Membrane depolarization assay.

The S. epidermidis ATCC 35984 and S. epidermidis 5 membrane potential was measured using an established protocol (19). The cells were resuspended in 5 mM HEPES to 1 × 10^8^ CFU/mL, mixed with 0.4 μM 3,3′-dipropylthiadicarbocyanine iodide (3,3-dipropylthiadicarbocyanine iodide [DiSC3-5]; Sigma-Aldrich, USA) and 0.1 M KCl, and then detected with a microplate reader at an excitation wavelength of 622 nm and emission wavelength of 670 nm for 20 min. After that, 50 μL of C9 (0 to 128 μg/mL) or Triton X-100 (1%) was added, and the mixture was monitored continuously for 40 min.

### Membrane fluidity assay.

The S. epidermidis ATCC 35984 and S. epidermidis 5 were resuspended in PBS to 1.5 × 10^6^ CFU/mL, and 100 μL suspension containing 10 μM 6-dodecanoyl-*N*,*N*-dimethyl-2-naphthylamine (Laurdan, Sigma-Aldrich, USA) was added to a black microplate containing C9 (0 to 128 μg/mL) or benzyl alcohol. After incubation for 1 h, the fluorescence was detected at wavelengths of 350 nm for excitation and 435 nm for emission. The Laurdan generalized polarization (GP) was calculated: GP = (I_435_ − I_490_)**/**(I_435_ + I_490_), where I_435_ and I_490_ show fluorescence intensity at 435 nm and 490 nm, respectively (36).

### ROS measurements.

2,7-dichlorodihydrofluorescein diacetate (DCFH-DA, Yuanye Bio-Technology, China) was applied to monitor the levels of ROS in S. epidermidis ATCC 35984 and S. epidermidis 5 (59). The bacterial suspension (1 × 10^8^ CFU/mL) was incubated with DCFH-DA (2 μM) for 30 min, washed, resuspended in PBS, and then added to a black microplate containing C9 (0 to 32 μg/mL) and incubated for 1 h. The fluorescence intensity was detected at 488 nm excitation and 525 nm emission wavelength.

### Confocal microscopy assay.

The distribution of FITC-C9 in S. epidermidis ATCC 35984 was observed by CLSM (60). The bacteria (1 × 10^8^ CFU/mL) were incubated with FITC-C9 (1× MIC) for 1 h and observed under the CLSM (Olympus, FV1000, Japan).

### DNA binding assay.

The interaction between C9 and the genomic DNA of S. epidermidis was determined by a DNA gel retardation assay (61). S. epidermidis genomic DNA was extracted using a bacterial genomic DNA extraction kit (TaKaRa, China). An equal volume of DNA (39.8 ng/μL, optical density at 260 nm [OD260]/OD280_nm_, 1.8) was mixed with C9 (0 to 64 μg/mL) and incubated for 1 h and then electrophoresed in 1% agarose (120 V, 30 min). DNA migration was observed in a gel imaging system and photographed.

### Transcriptome analysis.

In brief, the S. epidermidis ATCC 35984 overnight cultures were diluted 1:100 and grown in TSB to an OD_600_ of 0.5 and then treated with C9 (4 μg/mL) or PBS for 3 h (62). After incubation, cells were harvested and frozen in liquid nitrogen and stored at **−**80°C. RNA extraction, RNA-Seq library construction, and RNA sequencing were performed by the Novogene Corporation (Beijing, China). Three biological replicates were set for each group.

The quality and quantity of RNA were detected using an Agilent 2100 bioanalyzer and sequenced with an Illumina NovaSeq 6000 high-throughput sequencing platform. The raw data were quality-control filtered to obtain clean reads for subsequent analysis and compared with the reference genome of S. epidermidis ATCC 14990 (CP035288.1) using Bowtie 2 software. After gene expression quantification was completed, the differentially expressed genes were screened according to the criteria of *P < *0.05 and log_2_ (fold change) > 0 (63).

### Quantitative real-time PCR analysis (qRT-PCR).

The expression levels of seven genes were examined by qRT-PCR to validate the RNA-Seq results. The bacterial samples were prepared according to transcriptome, total RNA was extracted using the M5 EASY spin plus kit (Mei5 Biotechnology, China), and 1 μg RNA was reverse transcribed into cDNA with a PrimeScript RT reagent kit (TaKaRa, China). According to the SYBR premix *Ex Taq* kit (TaKaRa) protocol, the reactions were run on an ABI 7300 real-time PCR system. Gene expression levels were normalized using the S. epidermidis *gyrB* gene (34), and relative expression levels were calculated using the 2^–ΔΔ^*^CT^* analysis method. The primers are listed in Table S5.

### Hemolysis assays.

The hemolytic activity of C9 was evaluated based on a previous study (64). Freshly collected human blood (heparinized) from healthy donors was centrifuged at 3,000 rpm and washed with 1× PBS until the supernatant was clear. Then 100 μL 4% (vol/vol) red blood cell suspension was mixed with the different concentrations of C9 (4 to 512 μg/mL, 100 μL); the same volume of 1× PBS and 0.1% Triton X-100 were used as the negative and positive control, respectively. After being incubated for 1 h, the supernatant (100 μL) was transferred to a new 96-well plate to record the absorbance at 540 nm. The percentage of hemolysis was determined using the following formula: hemolysis (%) = (A _Treated_ − A_PBS_/A_0.1% Triton X-100_ − A_PBS_) × 100%.

### *In vivo* toxicity experiments.

BALB/c female mice (6 to 8 weeks old) were purchased from SPF Biotechnology Co., Ltd. (Beijing, China). All mice were housed in an independently ventilated cage system and given adequate food and water. All animal studies were conducted according to the guidelines of the animal welfare system and approved by the Animal Ethics and Use Committee of Guizhou Medical University (project no. 2000057).

The LD_50_ of C9 in mice was determined using previously described methods (65). A total of 20 mice were randomly divided into 4 groups and intraperitoneally (i.p.) injected with C9 (0 to 128 mg/kg, 0.2 mL). The survival rate, abnormal behavior, and body weight of mice in each group were recorded within 7 days. To evaluate the toxicity of C9 on the liver and kidney of mice (53), 0.2 mL C9 (64 mg/kg) or saline was administered i.p. to normal mice (*n* = 5 mice per group), which were sacrificed for blood biochemical analyses and histopathological observation after 24 h. Serum levels of alanine aminotransferase (ALT), aspartate aminotransferase (AST), and albumin (ALB) were determined to assess liver function, and serum creatinine (Cr) and urea (UREA) were measured to evaluate nephrotoxicity. For histopathological observation, liver and kidney tissue inflammatory changes were observed in a three-dimensional (3D) panoramic scanner (3D HISTECH Panasonic 250, Hungary).

### Mouse peritoneal model of infection.

A mouse model of acute bacterial infection was established by i.p. injection of S. epidermidis (66). In brief, S. epidermidis ATCC 35984 was injected i.p. to each group of mice (*n* = 5 mice per group) at a dose of 0, 5 × 10^8^, 1 × 10^9^, or 3 × 10^9^ CFU in 200 μL saline, and the mice were observed for 7 days. The mice were injected i.p. with S. epidermidis ATCC 35984 (1 × 10^9^ CFU) and treated with C9 (10 mg/kg, i.p., 0.2 mL), C9 (10 mg/kg, intravenously [i.v.], 0.2 mL), vancomycin (10 mg/kg, i.v., 0.2 mL), or saline 2 h after infection. The survival of the mice was recorded daily for 7 days.

### Statistical analysis.

Statistical analysis was performed using an independent sample *t* test or a one-way analysis of variance (ANOVA) using a Bonferroni test to determine statistical differences between groups. The significance level was set as follows: NS, not significant; *, *P < *0.05; **, *P* < 0.01; ***, *P* < 0.001. All statistical tests were two-sided, and data represent means ± standard deviation (SD). All statistical data analyses were performed using GraphPad Prism software (version 8.0). All experiments were performed in triplicate, except where indicated.

### Ethical approval.

All materials used in this study were approved for use by the Institutional Review Board, and all methods/experiments were conducted in accordance with the guidelines approved by the Ethics Committee of Guizhou Medical University, China.

### Data availability.

The sequencing data in the article have been deposited at the National Center for Biotechnology Information under SRA no. PRJNA886054.
