# Supplementary material for: Cec4-Derived Peptide Inhibits Planktonic and Biofilm-Associated Methicillin Resistant Staphylococcus epidermidis
Source: Microbiol Spectr. 2022 Dec 1;10(6):e02409-22. doi: 10.1128/spectrum.02409-22 (PMC9769716; doi:10.1128/spectrum.02409-22)
Supplement: Supplemental file 1 — Supplemental material. Download spectrum.02409-22-s0001.pdf, PDF file, 0.4 MB [file spectrum.02409-22-s0001.pdf]

Table S1. Strain information and MIC values of *S. epidermidis*

Strain	Origin	MIC(μg/mL)		
		C9	Oxacillin	Vancomycin
SE ATCC 35984	-	8	0.5	2
SE 1	Blood	8	2	2
SE 2	Urine	8	>4	2
SE 3	Blood	8	0.5	2
SE 4	Secretion	8	>4	2
SE 5	Biopsy tissue	8	1	2
SE 6	Central Catheter	8	1	2
SE 7	Blood	8	2	2
SE 8	Sputum	8	>2	1
SE 9	Urine	8	>4	2
SE 10	Blood	8	>2	4
SE 11	Secretion	8	≤0.25	2
SE 12	Blood	8	>2	2
SE 13	Central Catheter	8	>4	1
SE 14	Blood	8	>4	2
SE 15	Biopsy tissue	8	≤0.25	2
SE 16	Blood	8	>4	1
SE 17	Blood	8	>2	1
SE 18	Blood	8	>4	2
SE 19	Urine	8	≤0.25	1
SE20	Blood	8	>4	2
SE 21	Central Catheter	8	>4	2
SE 22	Urine	8	>4	2
SE 23	Blood	8	≤0.25	2
SE 24	Central Catheter	8	>4	2
SE 25	Blood	8	>4	2
SE 26	Dialysis solution	8	>4	1
SE 27	Blood	8	>4	1
SE 28	Blood	8	>4	2
SE 29	Blood	8	>4	2
SE 30	Urine	8	≤0.25	1
SE 31	Blood	8	≤0.25	2
SE 32	Urine	8	>4	≤0.5
SE 33	Ascites	8	>2	2
SE 34	Central Catheter	8	>2	1

TABLE S2 The synergy analysis of C9 with clinical antibacterial agents.

Strain	MIC				FICI		
	C9	Van	Cip	Oxa	Van	Cip	Oxa
SE 5	8	2	1	1	1<br>（4/1）	1<br>（4/0.5）	0.75<br>（2/0.5）
SE ATCC35984	8	2	0.5	0.5	1<br>（4/1）	0.75<br>（2/0.25）	0.625<br>（4/0.0625）

TABLE S3 Genes with significantly altered transcript levels after C9 treatment

Gene name	Description	log 2 Fold change	Corrected *P* value
Cell wall			
EQW00_RS05660	penicillin-binding protein	+0.47	3.62×10^{-2}
dltD	D-alanyl-lipoteichoic acid biosynthesis protein DltD	+0.81	3.13×10^{-4}
vraX	C1q-binding complement inhibitor VraX	+1.22	4.63×10^{-2}
Membrane protein			
EQW00_RS02955	ABC transporter permease	+1.23	1.03×10^{-6}
EQW00_RS02960	ABC transporter ATP-binding protein	+1.58	7.17×10^{-4}
EQW00_RS01850	anaerobic C4-dicarboxylate transporter	+1.19	3.51×10^{-2}
Electron Transport			
EQW00_RS02315	NAD (P)H-dependent oxidoreductase	+0.34	4.81×10^{-2}
EQW00_RS02195	zinc-dependent alcohol dehydrogenase family protein	+0.91	3.33×10^{-2}
EQW00_RS08715	cytochrome ubiquinol oxidase subunit I	+0.43	7.75×10^{-3}
Ribosome			
rpsO	30S ribosomal protein S15	+0.93	3.40×10^{-2}
rpsJ	30S ribosomal protein S10	+0.58	3.73×10^{-2}
EQW00_RS11120	50S ribosomal protein L10	+0.96	4.81×10^{-2}
EQW00_RS04105	type B 50S ribosomal protein L31	+0.91	1.27×10^{-2}

EQW00_RS11095	ribosomal L7Ae/L30e/S12e/Gadd45 family protein	+0.57	3.43×10^{-2}
Oxidative stress			
tpx	thiol peroxidase	+0.89	8.42×10^{-3}
EQW00_RS09670	organic hydroperoxide resistance protein	+1.24	2.10×10^{-3}
EQW00_RS11790	general stress protein	+1.49	3.77×10^{-2}
Transcription Factors and Regulators			
ccpA	catabolite control protein A	+0.88	4.30×10^{-2}
EQW00_RS01635	Crp/Fnr family transcriptional regulator	+0.84	7.87×10^{-3}
EQW00_RS06770	transcriptional repressor	+0.75	6.10×10^{-3}
spxA	transcriptional regulator Spx	+1.68	4.92×10^{-2}
EQW00_RS02455	GntR family transcriptional regulator	+0.49	1.60×10^{-2}
EQW00_RS03200	MurR/RpiR family transcriptional regulator	+1.06	1.00×10^{-2}
EQW00_RS02830	MarR family transcriptional regulator	+0.88	3.58×10^{-2}
EQW00_RS10285	DeoR/GlpR transcriptional regulator	+0.51	3.77×10^{-2}
Another			
gap	type I glyceraldehyde-3-phosphate dehydrogenase	+0.70	6.03×10^{-2}
argF	ornithine carbamoyltransferase	+1.75	3.03×10^{-2}
arcC	carbamate kinase	+1.62	4.00×10^{-2}
ftnA	H-type ferritin FtnA	+1.08	2.08×10^{-4}
EQW00_RS05645	bifunctional 3-deoxy-7-phosphoheptulonate synthase	+0.73	1.48×10^{-2}
EQW00_RS09745	epsilon family phenol-soluble module	-1.17	4.69×10^{-2}
EQW00_RS09510	teichoic acid D-Ala incorporation-associated protein	-0.84	3.67×10-2
EQW00_RS06455	DEAD/DEAH box helicase	-0.45	1.33×10-2

TABLE S4 Amino acid sequence and physicochemical properties of antimicrobial peptides

Peptide	Sequence	MW (Da)	Charge	Isoelectric Point	GRAVY
C9	LWKIGKKIWRVGWNWR	2126.58	5	12.02	-0.725

TABLE S5 Primers used for RNA-seq was validated by qRT-PCR.

Target gene		The sequence of primers (5' to 3')	Amplicon (bp)
gyrB	F	TGGTCTGCGTTCATTTCACCAAGAC	248
	R	CTTGCCGATGTTGATGGTGCACA	
RS02960	F	AACTGGAATGTCTAGCAAAGAAGC	112
	R	TTGTTGTCCACCTGAAAGCATA	
RS06455	F	TAGGACAATCTCAAACTGGAACTG	100
	R	ACTACGATGGCTTGTGGCTCT	
dltX	F	ATGAAAGGAAAAGAGCCTTCTAACA	67
	R	ATAACAAAGTGATTAAGTAAGGCTT	
rpsO	F	ACCAGAAGTCCAAATCGCTGTA	116
	R	GACGACCTACCATTTTCAATAAACC	
dltD	F	CTTATTGGTGCGGGAGGTTC	292
	R	CATTGGGATGTTTTGCTTGTTC	
spxA	F	CTTGCCGTAAAGCGAAAGC	130
	R	TCGTCAGTTCCATCTTCAGTCAT	
vraX	F	ATGATTATCTACAGAAGAAATATAG	71
	R	ATGTTGTCAACGTCATTCTCTAGTA	

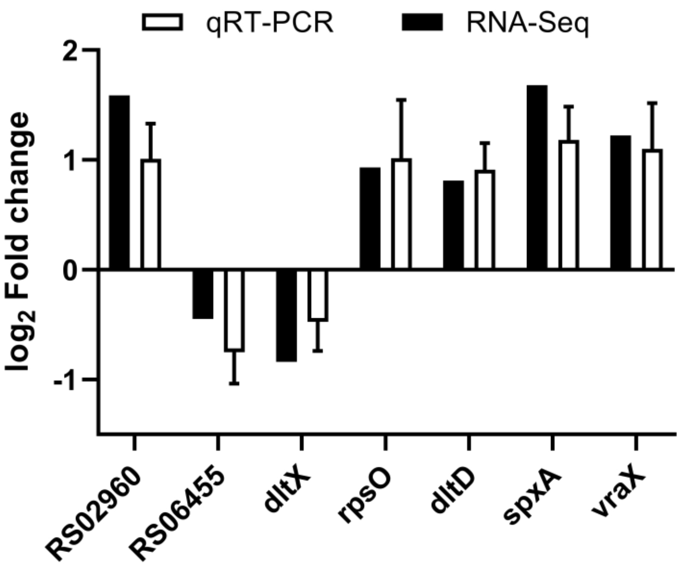

FIG S1 Comparison of seven gene expression levels between RNA-Seq and qRT-PCR.

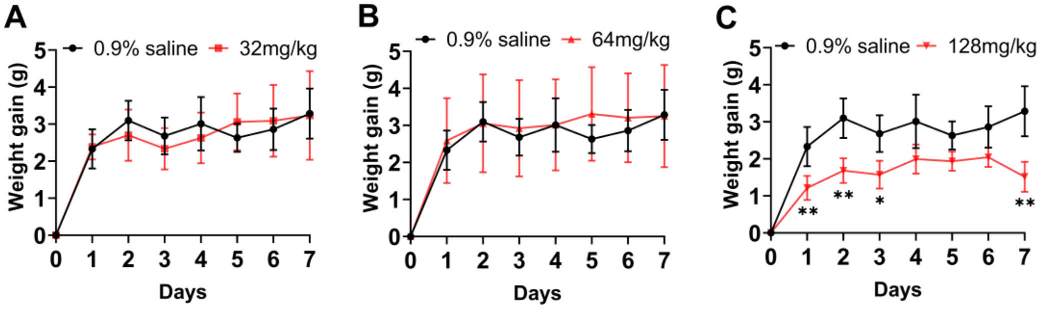

FIG S2 The weight changes after C9 intraperitoneal injection. (A-C) The weight gain of mice treated with C9 (32 mg/kg, 64 mg/kg, and 128 mg/kg). (C) The statistics reflect the daily weight gain of mice after intraperitoneal administration of C9 (128 mg/kg) compared to the 0.9% saline group. Data were analyzed by independent sample T-test. *$P < 0.05$, **$P < 0.01$.
